# Transforming Growth Factor-β Promotes Morphomechanical Effects Involved in Epithelial to Mesenchymal Transition in Living Hepatocellular Carcinoma

**DOI:** 10.3390/ijms20010108

**Published:** 2018-12-28

**Authors:** Mariafrancesca Cascione, Stefano Leporatti, Francesco Dituri, Gianluigi Giannelli

**Affiliations:** 1 Department of the Medical Sciences and Human Oncology, University Aldo Moro, 70124 Bari, Italy; 2 CNR Nanotec—Institute of Nanotechnology, 73100 Lecce, Italy; 3 National Institute of Gastroenterology “S. de Bellis” Research Hospital, 70013 Castellana Grotte (Ba), Italy; francesco.dituri@irccsdebellis.it (F.D.); gianluigi.giannelli@irccsdebellis.it (G.G.)

**Keywords:** TGF-β1, epithelial to mesenchymal transition (EMT), atomic force microscopy (AFM)

## Abstract

The epithelial mesenchymal transition (EMT) is a physiological multistep process involving epithelial cells acquiring a mesenchymal-like phenotype. It is widely demonstrated that EMT is linked to tumor progression and metastasis. The transforming growth factor (TGF)-β pathways have been widely investigated, but its role in the hepatocarcinoma EMT is still unclear. While the biochemical pathways have been extensively studied, the alteration of biomechanical behavior correlated to cellular phenotype and motility is not yet fully understood. To better define the involvement of TGF-β1 in the metastatic progression process in different hepatocarcinoma cell lines (HepG2, PLC/PRF/5, HLE), we applied a systematic morphomechanical approach in order to investigate the physical and the structural characteristics. In addition, we evaluated the antitumor effect of LY2157299, a TGF-βR1 kinase inhibitor, from a biomechanical point of view, using Atomic Force and Confocal Microscopy. Our approach allows for validation of biological data, therefore it may be used in the future as a diagnostic tool to be combined with conventional biomolecular techniques.

## 1. Introduction

The interest in cell mechanics studies is rapidly expanding within the cancer research community; the increasing attention to involvement of biomechanical properties within cancer biology investigations arises from the strict relationship between mechanics and intracellular biological events. In fact, cell mechanics play a central role in defining cell morphology and functions, thus understanding the mechanical properties of cells is essential to studying their physiology. In addition, many experimental works have demonstrated that cell mechanics are closely related to alterations in cytoskeleton structures, therefore changes in cell structure and mechanics, as well as alterations of the cellular response to external stimuli [1,2] are associated with the initiation and advancement of many diseases, such as cancer [3,4]. Particularly, the cytoskeletal architecture and mechanical behavior change in tumor cells: In vitro experiments have shown epithelial cancer cells are characterized by a stiffer extracellular matrix structure than that of healthy ones [5,6], whereas cell elasticity changes between different phases of cancer progression. Several works demonstrate that the cellular stiffness decreases when cancer cells transform from the not invasive to malignant phenotype, [2,7,8,9].

The Epithelial to Mesenchymal Transition (EMT) is a physiological multistep process involving epithelial cells acquiring an invasive phenotype; this process plays a central role in embryogenesis [10], but it is also leads to negative outcomes in cancer disease [11]. Indeed, during EMT, epithelial benign tumor cells lose their apical-basal polarity and their typical architecture, adopting a mesenchymal-like phenotype. As a result of these morphological changes, cells are able to detach themselves from the basement membrane and they may infiltrate the surrounding tissue [12,13].

EMT can be stimulated by several soluble signals, among which transforming growth factor (TGF)-β has been recognized as one of the main promoters of EMT [14,15,16]. TGF-β is a growth-inhibitory cytokine, and it is the prototype of the family, including 33 members of peptide growth factors. The TGF-β family is ubiquitous, multifunctional and essential for survival. In fact, it has a key role in regulation of growth, development of tissues, and in maintenance of homeostasis [17,18]. A unique gene encodes three different isoforms of mammalian TGF-β, which are secreted in ECM as a latent protein complex: TGF-β1, -β2 and -β3, but -β1 is the most abundant and universally expressed. When the TGF-β heterodimer complex switches in active form, it binds with type I and type II receptors (TβR1 and TβR2) which are present on the cell surface. The intracellular domains of TβR1-2 contain serine/threonine kinase; upon binding, serine/threonine kinase of TβR2 phosphorylates the serine/threonine kinase of the TβRI, which in turn phosphorylates a family of transcriptional factors, namely Smads [19]. The activated complex formed by Smad2 and Smad3, associated with Smad4, translocates into the nucleus where it activates an EMT-inducing transcription factor, such as Snail/Slug, ZEB1/2 or Twist [20,21]. Their activation inhibits expression of epithelial markers and activates mesenchymal ones [22]. Recently, Galunisertib (LY2157299) was reported as an inhibitor of TGF-β receptor I kinase, which abrogates activation of the canonical EMT pathway [23,24,25]. Some experimental research demonstrated the efficient inhibition of p-Smad2 expression as well as invasion, but not proliferation, in three hepatocarcinoma cells (HCC) models in vitro [23,26,27,28].

The acquisition of motility and invasive capacity, needed for metastasis, requires a dramatic rearrangement of the actin cytoskeleton and alters physiological interaction of cells with the extracellular matrix (ECM). This induces a change in the cytomechanical properties, such as cell membrane elasticity. The cell membrane is involved in several processes, including signal sensing, shape, deformability, motility, division, and adhesion. It exerts and responds to forces, due to its interaction with the cytoskeleton and motor proteins [29].

The mechanical properties of the cell membrane, especially its elastic modulus, play a fundamental role in the dynamics of these processes. Therefore, alteration of this parameter can be used as marker of the cellular physiological state. 

Atomic Force Microscopy (AFM) is a powerful tool to quantify cell mechanical properties. Elastic properties, expressed in term of Young’s modulus (E), can be determined by analyzing the interaction between the AFM probe and the cell surface [2,30]. Recently, many efforts are addressed to assess biomechanical effects induced by cancer disease progression.

In addition, the role of TGF-β has been investigated in different kind of cancer cells: Kidney [31], alveolar [32], mammalian [33], liver [34], pancreatic [35], and breast [36].

The aim of this work is the characterization of biomechanical effects induced by TGF-β1 and its specific receptor TβR1 inhibitor (Galunisertib, LY2157299) in EMT progression of three different epithelial hepatocarcinoma cells (HCC) lines, previously studied [37]:

- Hep3B and PLC/PRF/5 cell lines, having an epithelial-like phenotype. These cell lines do not secrete TGF-β1.
- HLE cell line, having a mesenchymal-like phenotype. These cells produce autocrine TGF-β1.

The role of TGF-β1 in EMT was investigated by stimulating Hep3B and PLC/PRF/5, whereas the therapeutic effects of LY2157299 was studied in HLE cells and confirmed in Hep3B cells, which were previously stimulated with TGF-β1.

In summary, the biomechanical effects were evaluated by means of AFM to quantify Young’s modulus and the correspondent morphological perturbations were investigated by confocal analysis. This rigorous approach permits better understanding of the biological phenomena and correlates morphomechanical aspects to specific cell molecular pathways.

## 2. Results

The morphometric variations, due to the treatments, were evaluated by analyzing confocal acquisitions on different focal planes (Z stack mode).

**Figure 1:**
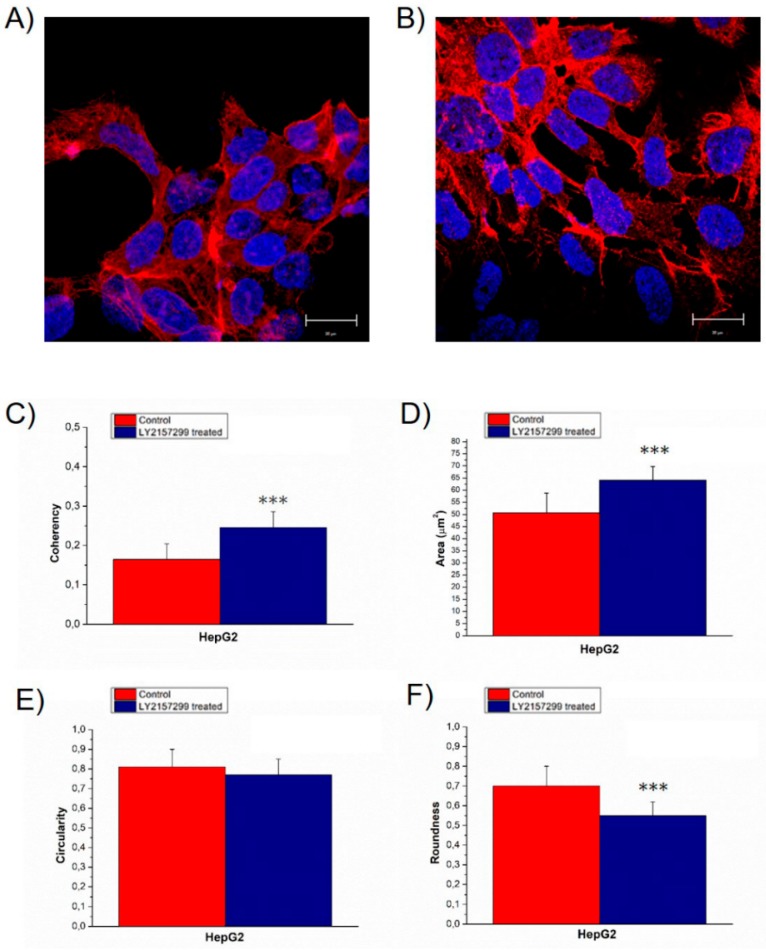
Representative confocal images of HepG2^CTR^ (**A**) and transforming growth factor (TGF)-β treated for 48 h HepG2^TGF-β1^ (**B**), the scale bar in the figures correspond to 20 µm. In the panel, the value of F-actin coherency (**C**), nuclear area (**D**), circularity (**E**) and roundness (**F**) were reported. The analysis of F-actin and nuclear morphology of control and TGF-β1 treated HepG2 cells, were performed by using ImageJ software. Results were statistically significant for *p* < 0.005 (indicated as ***).

HepG2 control cells grew in pile up structures formed by a few overlapped layers, in which cells were strictly adherent (Figure 1A). After TGF-β1 treatment, actin assembly clearly changed inducing a more elongated cellular shape; the cells were less adherent each other, and at the periphery of the cytoskeleton, they exhibited thin cytoplasmic projections that extend beyond the leading edge (Figure 1B).

The alterations in actin organization were expressed in term of coherency (Figure 1C), estimated by analysis of the confocal images using ImageJ software. This parameter expresses the local orientation in a selected region of a wall image [38,39]; it ranges from 0 to 1, indicating isotropic orientation and highly oriented structures respectively.

This value increased from (0.16 ± 0.04) to (0.25 ± 0.04) after TGF-β1 external stimulation; in both cases this value indicated a strong anisotropy in term of actin orientation fibers, but HepG2^TGF-β1^ exhibited a less disordered actin network.

In addition, the TGF-β1 external exposure induced an increase in nuclear size from (25 ± 4) to (32 ± 3) µm^2^ (Figure 1D). The alterations in nuclear morphology induced by TGF-β1 treatment were quantified by means of specific morphology descriptors: Circularity (Figure 1E) and roundness (Figure 1F). Roundness of HepG2^TGF-β1^ decreased from (0.7 ± 0.1) to (0.55 ± 0.07), whereas the circularity did not show significant changes.

**Figure 2:**
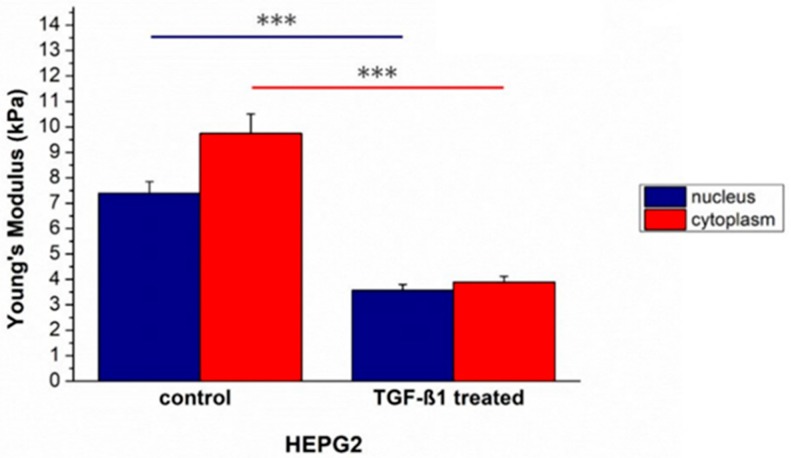
The Young’s modulus values with standard deviation, calculated from the nuclear region and the cytoskeletal area respectively, were reported for untreated HepG2^CTR^ and TGF-β1 treated HepG2^TGF-β1^ cells. Results were statistically significant for *p* < 0.005 (indicated as ***).

After morphological changes investigation in HepG2^CTR^ cells induced by TGF-β1 external exposure, the mechanical properties were assessed and quantified in term of elasticity. Young’s modulus values decreased: It was (7.4 ± 0.5) kPa for the nucleus and (9.7 ± 0.8) kPa for the cytoplasm in HepG2^CTR^, while in in HepG2^TGF-β1^ the values changed, becoming (3.6 ± 0.2) and (3.9 ± 0.2) kPa for the nuclear and cytoplasmic regions, respectively (Figure 2). HepG2^TGF-β1^ cells showed a comparable Young’s modulus, within the associated measurement error, in the two main cellular compartments analyzed.

**Figure 3:**
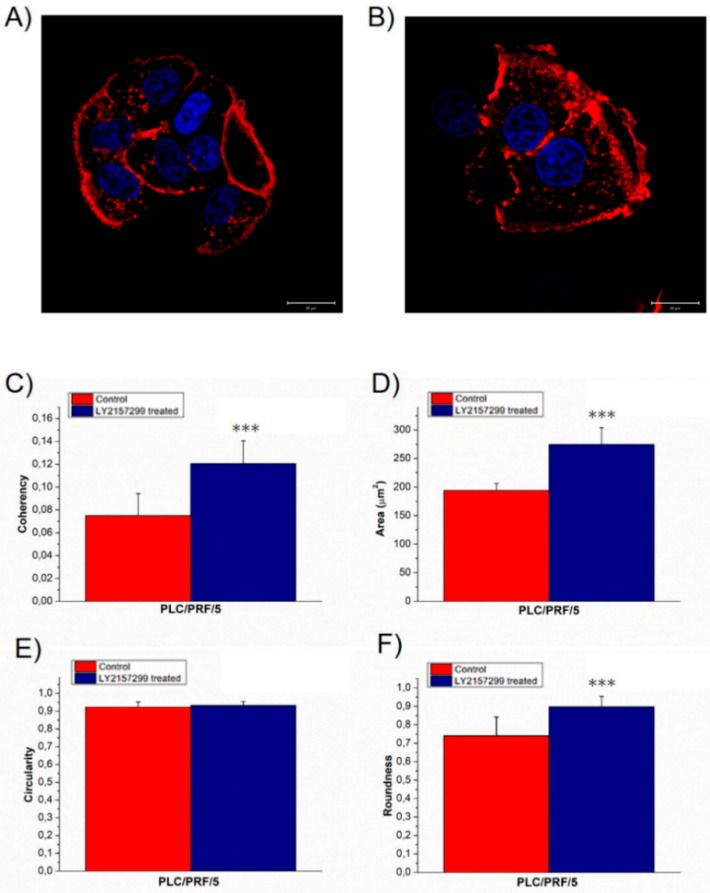
Representative confocal images of PLC/PRF/5^CTR^ (**A**) and TGF-β treated for 48 h, PLC/PRF/5^TGF-β1^ (**B**); the scale bar in the figures correspond to 20 µm. In the panel the value of F-actin coherency (**C**), nuclear area (**D**), circularity (**E**) and roundness (**F**) were reported. The analysis of F-actin and nuclear morphology of control and TGF-β1 treated PLC/PRF/5 cells, by using ImageJ software. Results were statistically significant for *p* < 0.005 (indicated as ***).

In order to confirm the obtained results due to external TGF-β1 stimulation, morphomechanical alterations were also evaluated on another epithelial-like HCC cell line: PLC/PRF/5. Confocal acquisitions showed a small perturbation in treated cell morphology compared to the negative control (Figure 3A,B). In particular, the actin fibers in untreated and treated cells were strongly disorganized, but the TGF-β1 exposure slightly induced less anisotropy; in details, the coherency values (0.08 ± 0.02) in PLC/PRF/5^CTR^ turned into (0.12 ± 0.02) for PLC/PRF/5^TGF-β1^ (Figure 3C). The analysis conducted on nuclei recorded a significant area increase, from (194 ± 12) to (275 ± 29) µm^2^ (Figure 3D). In addition, nuclear circularity remained unaltered (Figure 3E) and roundness increased from (0.7 ± 0.1) to (0.90 ± 0.06) for control and treated cells, respectively (Figure 3F). These data indicated that TGF-β1 external stimulation did not induce the formations of protrusions on the nuclear membrane and enhanced the round shape of nuclei.

**Figure 4:**
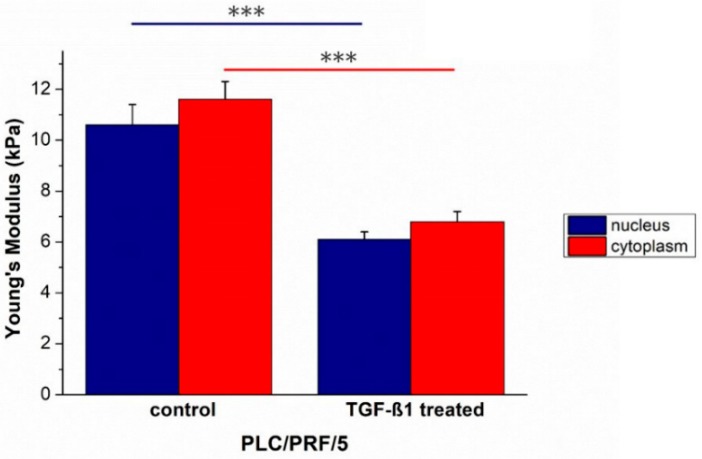
The Young’s modulus values with standard deviation, calculated from the nuclear region and the cytoskeletal area respectively, were reported for untreated PLC/PRF/5^CTR^ and TGF-β1 treated PLC/PRF/5^TGF-β1^ cells. Results were statistically significant for *p* < 0.005 (indicated as ***).

As obtained for Hep3B, TGF-β1 treatment induced an increase in term of elasticity in the main cellular components of PLC/PRF/5. In fact, Young’s modulus values decreased from (10.6 ± 0.8) to (6.1 ± 0.3) kPa for the nuclei, and from (11.6 ± 0.7) to (6.8 ± 0.4) kPa for the cytoplasmic region (Figure 4).

**Figure 5:**
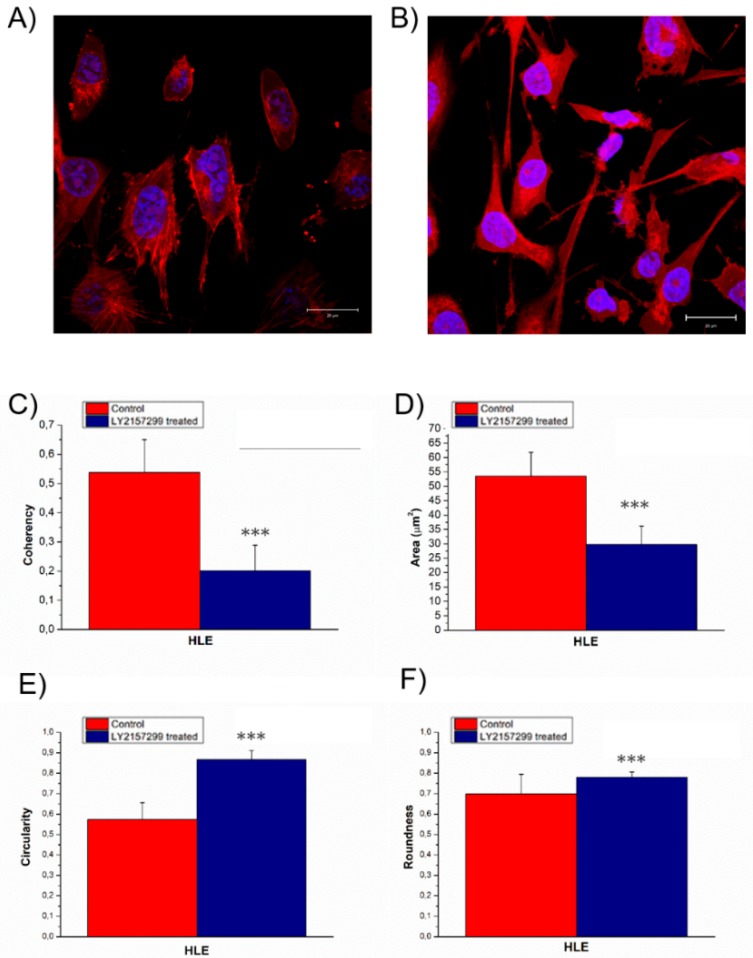
Representative confocal images of HLE^CTR^ (**A**) and Galunisertib HLE^LY2157299^ treated for 48 h (**B**), the scale bar in the figures correspond to 20 µm. In the panel the value of F-actin coherency (**C**), nuclear area (**D**), circularity (**E**) and roundness (**F**) were reported. Analysis of F-actin and nuclear morphology of control and Galunisertib treated HLE cells, by using ImageJ software. Results were statistically significant for *p* < 0.005 (indicated as ***).

HLE grown isolated exhibited the morphological characteristics typical of the mesenchymal phenotype (Figure 5A). The untreated cells displayed aligned actin fibers, whereas after 48 h Galunisertib incubation, the actin filaments appeared strongly disorganized (Figure 5B). In fact, the coherency values (Figure 5C) decreased from (0.5 ± 0.1) to (0.20 ± 0.09). The nuclei after treatment became small and they presented irregularity on their surface. In detail, the nuclei area changed from (27 ± 4) to (14 ± 3) (Figure 5D), the circularity (Figure 5E) switched from (0.57 ± 0.08) to (0.87 ± 0.04), although the roundness was unmodified: (0.7 ± 0.1) and (0.78 ± 0.03) for the negative control and Galunisertib treated, respectively (Figure 5F). 

**Figure 6:**
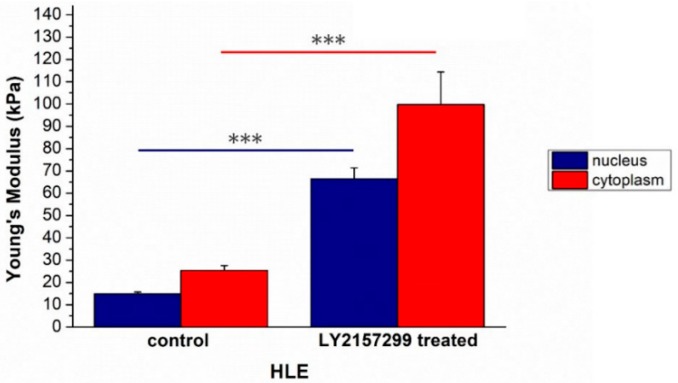
The Young’s modulus values with standard deviation, calculated from the nuclear region and the cytoskeletal area respectively, were reported for HLE^CTR^ and HLE^LY2157299^ cells. Results were statistically significant for *p* < 0.005 (indicated as ***).

Also, in this case, the morphological alterations were correlated to Young’s modulus modification; its value changed after treatment from (14.9 ± 0,8) to (66 ± 1) kPa for the nuclei and from (25 ± 2) to (100 ± 15) kPa for the cytoskeletal region (Figure 6).

**Figure 7:**
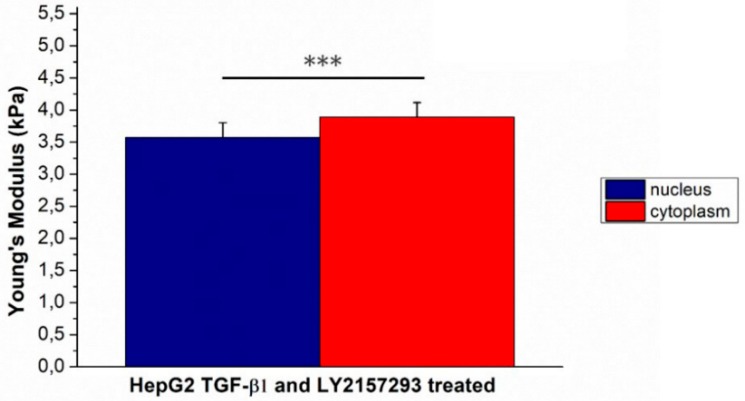
The Young’s modulus values with standard deviation, calculated from the nuclear region and the cytoskeletal area respectively, were reported for Galunisertib and TGF-β1 concurrently treated HepG2 cells. Results were statistically significant for *p* < 0.005 (indicated as ***).

Effect induced by Galunisertib on mechanical properties was also assessed HepG2 previously stimulated with TGF-β1. As expected, Young’s modulus of HepG2^TGF-β1^ decreased after Galunisertib treatment: (6.74 ± 0.33) for nuclear region and (7.57 ± 0.42) for cytoplasmic one (Figure 7).

## 3. Discussion

Although cancer is one of the leading causes of death, the mechanisms underlying tumor progression are still unclear today. Numerous biological studies have been carried out to investigate the biochemical pathways at the base of this progression, however our understanding is not yet sufficient. Within the same pathology, as in the case of hepatocarcinoma, identifying clinical-diagnostic tools is complicated due to the strong heterogeneity of the processes involved. The use of a complementary approach to classical biological techniques is necessary for a deeper understanding of the mechanisms involved in tumor progression.

Numerous studies have focused on the alteration in mechanical behavior in cancer cells in respect to their normal counterparts, highlighting that cell softness has been shown to increase during cancer progression [2,40,41,42]. 

In this study, to better understand the HCC progression, a morphomechanical approach was adopted, highlighting morphological and cytoskeletal differences among three HCC cell lines. HepG2, PLC/PRF/5 and HLE were chosen as representative of different EMT phenotypes due to specific genes and protein expression profiles, as reported in our previous work [37].

HepG2 and PLC/PRF/5 showed an epithelial-like phenotype, thus we assessed the effects induced by TGF-β1 exogenous stimulation, which is directly involved in hepatocarcinoma progression and promotes the EMT [43,44,45].

In vitro TGF-β1 stimulation affected HepG2 morphology in terms of cellular shape, adhesion, actin fiber orientation. The more elongated shape, the loss of cell-cell adhesions and the appearance of cytoplasmic projections in HepG2^TGF-β1^ suggested the acquisition of a different phenotype having a bigger migratory potential. The AFM analysis confirmed our hypothesis, showing an overall reduction of cellular Young’s modulus. Specifically, this effect mainly involved the cytoskeletal region, and HepG2^TGF-β1^ cells lost their region-specific mechanical properties; in fact, the Young’s modulus of the cytoskeletal area appears to be the same as the nucleus. These results suggested that the whole cellular body took part in the mechanical response, with a rearrangement that involved not only the cytoskeletal elements but also the nuclear region.

In addition to that, the analysis performed on confocal images revealed that TGF-β1 external exposure induced an increase in nuclear size and alterations in nuclear morphology: Nuclei lost their round shape becoming more elongated, but they did not show protrusions on the surface; this speculation was a consequence of circularity and roundness analysis [46,47,48].

These results are in accordance to literature data, according to which, nuclei of breast cancer epithelial cells varied in shape, becoming larger than the healthy ones [49,50]. A further evidence of the migratory potential for HepG2 cells in response to TGF-β1 stimulus, was the increase in the coherency parameter value due to a higher degree of actin fiber orientation. In fact, as reported in literature, when stress fibers were aligned to each other, this configuration is needed to promote cell migration in surrounding tissues [51,52].

The morphomechanical HepG2 behavior reflects the biochemical evidences concerning the stimulation of TGF-β1 pathways. Our recent study, demonstrates that the TGF-β1 (5 ng/mL) treatment induced a canonical EMT in HepG2^TGF-β1^, resulting in a decrease of epithelial gene expression (*CDH1* and *KRT18*) and in an up-regulation of mesenchymal ones (*CDH2*, *VIM*, *CXCR4*, *SNAI-1* and *-2*); thus an enhancement of ability to migrate and invade was linked [37]. Unlike what has been observed for HepG2, PLC/PRF/5 stimulated by TGF-β1 underwent partial/incomplete EMT because only *CDH2*, *VIM*, *CXCR4* were upregulated and the expression of some epithelial-related genes (*EPCAM*, *CD133*) was not only maintained but even increased [37].

Our confocal analysis showed that PLC/PRF/5^TGF-β1^, unlike HepG2^TGF-β1^, maintained cell–cell adhesions; they did not exhibit significant alterations in cell morphology and their nuclei acquired a more rounded shape. At the same time, as demonstrated for HepG2, stimulation with TGF-β1 induced an increase in nuclear area and a less disordered actin fibers network.

In accordance with reported biological evidence, by means of force spectroscopy analysis conducted by AFM, Young’s modulus of PLC/PRF/5 decreased, suggesting an enhancement of migratory abilities.

In summary, our results as obtained for HepG2 cell line are statistically significant and relevant; in fact, the percentage of Young’s modulus alteration was 51.4% for the nuclear region and 62.9% for the cytoplasmic one. In the case of TGF-β1 treated PLC/PRF/5 the percentage of Young’s modulus alteration was 42.5% and 41.4% for the nuclear and cytoplasmic area, respectively.

The morphomechanical changes induced by exogenous TGF-β1 exposure in HepG2 and PLC/PRF/5 confirmed its role in the tumorigenesis enhancement. For these reasons, TGF-β1 could be a potential target in therapeutic strategies, also taking into account that although HCC is one of the most lethal cancers [53], there are very few therapeutic options at the advanced stage.

In recent works, it was demonstrated that efficient inhibition of the TGF-β receptor I kinase specifically downregulates the phosphorylation of Smad2, as well as invasion in different HCC in vitro models [27,54]. Moving from these observations, we adopted our morphomechanical approach to investigate the effects of LY2157299 on a HLE mesenchymal-like cell line, characterized by autocrine production of TGF-β [27,37].

HLE^LY2157299^ displayed strongly disorganized actin filaments compared to the negative control, in which stress fibers were well aligned each other, as confirmed by coherency values relative to confocal acquisitions. Galunisertib treatment induced modifications in nuclear morphology above all in terms of circularity, suggesting an effective role in tumorigenesis inhibition. In fact, in metastatic cells, the nuclei are bean-shaped or segmented, in opposition to the classical spherical shape, thus they may develop higher morphological flexibility [55,56]. As expected, Galunisertib treatment also affected cell elasticity; the increased Young’s modulus of HLE^LY2157299^ reflected the loss of migratory capacity.

Finally, the therapeutic potential of Galunisertib was also tested in conditions that mimic the pathological state. As previously reported, TGF-β1 stimulated in HepG2 cells the canonical EMT, in which the Young’s modulus underwent a significant reduction. The addition of Galunisertib induced a reduction of invasive potential in HepG2^TGF-β1^ that corresponds to an increase in the Young’s modulus values.

Hepatocellular carcinoma is one of the most common malignancies worldwide, characterized by a high rate of death. Many efforts have been carried out to understand the biomolecular pathways involved in HCC progression. Currently, the rapid growth of nanotechnology in biological and biomedical fields benefits understanding in both biological information and mechanical properties; allowing expansion of the current knowledge on the cellular modifications associated with disease progression. In this context, the atomic force microscopy investigation has provided new insight to explain the TGF-β role in hepatocarcinoma EMT and to test from biomechanical point of view the effects of Galunisertib.

## 4. Materials and Methods

### 4.1. Cell Culture

Three different HCC cell lines, having different EMT phenotypes and TGF-β expression, were analyzed. Hep3B, PLC/PRF/5, and HLE cells were purchased by the European Collection of Cell Cultures (ECACC).

Hep3B and PLC/PRF/5 cells were grown in high-glucose Dulbecco’s modified Eagle’s medium (DMEM), while HLE cells were grown in Roswell Park Memorial Institute (RPMI) medium. Both media were supplemented with 10% (*v*/*v*) fetal bovine serum (FBS), 1% (*v*/*v*) l-glutamine, 1% (*v*/*v*) penicillin/streptomycin; all these products were provided by Sigma Aldrich (St. Louis, MI, USA).

### 4.2. Preparation of Samples

HepG2, HLE and PLC/PRF/5 cells were seeded in plastic Petri dishes (Corning, NY, USA) at a concentration of 10^5^ cell/mL and grown at 37 °C in a humidified atmosphere of 5% CO_2_ (*v*/*v*). After 24 h, some petri dishes of HepG2 and PLC/PRF/5 cells were stimulated for a whole 48 h with 5 ng/mL of human recombinant TGF-β1 (PEPROTEC, Rocky Hill, NJ, USA), specified as HepG2^TGF-β1^, PLC/PRF/5^TGF-β1^. HepG2 and PLC/PRF/5 cells without TGF-β1 external exposure (HepG2^CTR^, PLC/PRF/5^CTR^) represent the negative control.

HLE cells were treated with 10 µM Galunisertib (LY2157299) (Lilly, Indianapolis, IN, USA) for 48 h, indicated as HLE^LY2157299^, while the negative control is tagged as HLE^CTR^.

In addition, HepG2 cells were treated with 5 ng/mL of TGF-β1 for 1 h, and after this time 10 µM of Galunisertib was added. The cells stimulated this combined treatment were incubated for 48 h. Before performing AFM force-mapping measurements, the cells were washed with sterile phosphate buffer saline (PBS) solution and the medium was replaced in 3 mL of Lebovitz (L-15) medium (Sigma Aldrich).

The confocal experiments were carried out on fixed (glutaraldehyde at 0.25% in PBS for 10 min) HepG2^TGF-β1^, PLC/PRF/5^TGF-β1^, HLE^LY2157299^ cells and their control HepG2^CTR^, PLC/PRF/5^CTR^ and HLE^CTR^ respectively. After two washes with PBS, the cells were permeabilized with Triton (Sigma Aldrich) at 0.1% for 5 min and they were labeled with fluorescent markers: 1 µg/mL of phalloidin-TRITC (Sigma Aldrich) for 1 h to stain the actin cytoskeleton, and 1 μg/mL of Hoechst 33342 (Sigma Aldrich) for 5 min to label the nuclei.

### 4.3. Instruments

An advanced atomic force microscope (Bioscope Catalyst, Bruker Inc., Billerica, MA, USA) and a laser scanning confocal microscope (LSM 700, Zeiss, Oberkochen, Germany) were used at room temperature to perform the experiments. Both devices were mounted on an inverted optical microscope (Zeiss Observer Z1, Zeiss, Oberkochen, Germany). The whole system was placed on a base that acts as an insulator with respect to the environmental mechanical vibrations.

### 4.4. AFM Experiments

In this work, AFM experiments were carried out in force volume mode using the following parameters: Scan area (50 × 50) um^2^, Ramp rate 4.88 Hz, FV scan rate 0.03 Hz, Trigger Threshold 50 nm, Number of samples 512, Samples per line 128, Lines 128.

Acquisitions were performed using the V-shaped Bruker’s Sharp Microlever (MSNL, tip C); it consists of a sensitivity Silicon Nitride cantilever with a nominal elastic constant of 0.01 N/m. This value was accurately estimated using the thermal tune method [57], prior to performing each experiment.

From the acquired force-mapping images, 20 force-distance curves were extracted in correspondence of the nuclear area and 20 curves in the cytoplasmic region; this procedure was repeated on 20 cells.

The extracted curves were analyzed in order to estimate the local Young’s modulus E, which was evaluated by fitting the approach portion of force-distance curves with a modified Sneddon model:

$$\;-k_{c}\delta_{c}=\frac{2Etg\alpha }{\pi \left(1-\nu^{2}\right)}{\left(z-\delta_{c}\right)}^{2}\;$$

where *z* and *δ_c_* were the experimental loading data (height, cantilever deflection, respectively), *α* was half-angle of tip, *k_c_* was the elastic constant value of cantilever, and *ν* was the Poisson Ratio (assumed to be 0.5 for biological sample). In the fit algorithm, the contact point was treated as the fit variable and the adhesion forces were taken into account.

The extraction of indentation curves and their analysis were performed using Nanoscope Analysis software (Bruker Inc., Billerica, MA, USA). The Young’s modulus values, correspondent to the nuclear (E_nucleus_) and cytoplasmic (E_cytoplasm_) regions respectively, were calculated as average of values obtained from each extracted curve and its associated errors as standard deviation.

### 4.5. Confocal Experiments

The confocal images were obtained by exciting fluorescent proteins—Hoechst and phalloidin-TRITC with laser radiations at wavelengths of 405 and 555 nm. The Alpha Plan-Apochromat (ZEISS) 100× oil-immersion with 1.46 NA was used in the experiments and the acquisitions were performed in xy and in z-stack mode. The confocal acquisitions were analyzed by ZEN2010 software (ZEISS, Oberkochen, Germany) and morphometric quantifications (nuclear area, circularity and roundness of nuclei, coherency of F-actin) were performed on 15 cells, using the ImageJ 1.47 analysis software. Within the ImageJ suite, the quantifications of nuclei shape (i.e., circularity and roundness) were conducted using the particle analyser plugin within the ImageJ suite whereas the actin fiber coherency parameter was quantified using the OrientationJ plugin.

### 4.6. Statistical Analysis

The values obtained were expressed as mean values and their standard deviations. The results obtained were compared and furthermore a *t* test was used to test the statistical significance of results. Differences were considered statistically significant for *p*-values < 0.05. Data were analyzed and graphed using the OriginPro software (OriginLab version 8).

## 5. Conclusions

The heterogeneity of HCC makes it difficult to identify cellular targets useful for a therapeutic strategy that inhibits metastatic progression. The cell morphomechanical characterization may help to fully understand both the mechanisms underlying EMT and the effects induced by novel drugs acting on HCC. In this work, AFM was used to investigate the effects of TGF-β1 and its inhibition on different HCC cell lines, in terms of Young’s modulus alterations, correlating to the actin reorganization by confocal analysis. We found that the epithelial cell exhibits a more elastic behaviour after TGF-β1, suggesting increased migratory capability. Inversely, in mesenchymal cells we found an opposite effect after Galunisertib treatment. These results encourage development of antimetastatic HCC therapies based on inhibition of TGF-β1 receptors.
